# Tideglusib, a Non-ATP Competitive Inhibitor of GSK-3β as a Drug Candidate for the Treatment of Amyotrophic Lateral Sclerosis

**DOI:** 10.3390/ijms22168975

**Published:** 2021-08-20

**Authors:** Loreto Martínez-González, Claudia Gonzalo-Consuegra, Marta Gómez-Almería, Gracia Porras, Eva de Lago, Ángeles Martín-Requero, Ana Martínez

**Affiliations:** 1 Centro de Investigaciones Biológicas Margarita Salas, CSIC, Calle Ramiro Maétzu 9, 28040 Madrid, Spain; loretomg@cib.csic.es (L.M.-G.); graciapf@cib.csic.es (G.P.); 2 Instituto de Investigación en Neuroquίmica, Departamento de Bioquίmica y Biologίa Molecular, Facultad de Medicina, Universidad Complutense de Madrid, 28040 Madrid, Spain; clagon11@ucm.es (C.G.-C.); margom27@ucm.es (M.G.-A.); elagofem@ucm.es (E.d.L.); 3 Centro de Investigación Biomédica en Red Sobre Enfermedades Neurodegenerativas (CIBERNED), Instituto de Salud Carlos III, 28031 Madrid, Spain

**Keywords:** ALS, GSK-3β, Tideglusib, TDP-43

## Abstract

Amyotrophic Lateral Sclerosis (ALS) is the most common degenerative motor neuron disease in adults. About 97% of ALS patients present TDP-43 aggregates with post-translational modifications, such as hyperphosphorylation, in the cytoplasm of affected cells. GSK-3β is one of the protein kinases involved in TDP-43 phosphorylation. Up-regulation of its expression and activity is reported on spinal cord and cortex tissues of ALS patients. Here, we propose the repurposing of Tideglusib, an in-house non-ATP competitive GSK-3β inhibitor that is currently in clinical trials for autism and myotonic dystrophy, as a promising therapeutic strategy for ALS. With this aim we have evaluated the efficacy of Tideglusib in different experimental ALS models both in vitro and in vivo. Moreover, we observed that GSK-3β activity is increased in lymphoblasts from sporadic ALS patients, with a simultaneous increase in TDP-43 phosphorylation and cytosolic TDP-43 accumulation. Treatment with Tideglusib decreased not only phospho-TDP-43 levels but also recovered its nuclear localization in ALS lymphoblasts and in a human TDP-43 neuroblastoma model. Additionally, we found that chronic oral treatment with Tideglusib is able to reduce the increased TDP-43 phosphorylation in the spinal cord of Prp-hTDP-43^A315T^ mouse model. Therefore, we consider Tideglusib as a promising drug candidate for ALS, being proposed to start a clinical trial phase II by the end of the year.

## 1. Introduction

Amyotrophic lateral sclerosis (ALS) is a fatal neurodegenerative disorder characterized by progressive muscle paralysis due to motor neuron degeneration in the brainstem and spinal cord, causing premature death, mainly owing to respiratory failure. The etiopathogenesis of ALS is still unclear involving altered signaling pathways such as mitochondrial dysfunction, glutamate excitotoxicity, oxidative stress, protein aggregation, and neuroinflammation. Until now, no effective therapy to prevent the development and progression of the disease exists. Moreover, the heterogeneity of the disease both at the genetic and clinical levels had made the search for effective treatments a difficult task.

Despite the heterogeneity, about 97% of patients show cytoplasmic inclusions in motor neurons containing TDP-43, the protein product of *TARDBP* gene [1]. This suggests that similar pathogenic mechanisms may be present in different ALS subtypes. TDP-43 is a ubiquitously expressed, DNA/RNA binding protein, involved in RNA metabolism by regulating the transcription, stability, and transport of more than 600 mRNA [2]. TDP-43 is mainly located in the nucleus, but under pathological conditions, it translocates to the cytoplasm where it suffers different post-translational modifications including hyperphosphorylation, ubiquitination, and truncation, leading to formation of toxic C-terminal TDP-43 fragments and protein aggregation [3]. Phosphorylation of TDP-43 at Ser409/410 is one of the main pathological hallmarks of TDP-43 deposits observed in the vast majority of ALS patients [4]. Therefore, inhibitors of the protein kinases involved in TDP-43 phosphorylation may be innovative drug candidates for the future therapy of ALS and other TDP-43 proteinopathies.

Glycogen synthase kinase 3β (GSK-3β) has been identified as one of the protein kinases implicated in TDP-43 phosphorylation [5]. GSK-3β is a constitutively active multifunctional serine/threonine kinase involved in different physiological pathways, from gene expression to cell structure and proliferation. It has been reported its involvement in several diseases such as Alzheimer’s and Parkinson’s diseases (AD and PD), diabetes, and ALS [6]. In particular, GSK-3β expression and activity were found to be altered in *postmortem* spinal cords and frontal and temporal cortex of ALS patients [7,8,9,10]. Moreover, a correlation was established between GSK-3β levels and functional impairment in non-neuronal cells of ALS individuals, being a potential biomarker of disease progression [11].

All these findings are consistent with those reported in different cellular and animal models where GSK-3β inhibitors treatment increased cell viability and improved motor function. Particularly, TDP-43-positive stress granule formation, as well as C-terminal TDP-43 fragments, were reduced in the presence of a GSK-3β inhibitor in transfected cells [12]. Additionally, survival of human motor neurons derived from ALS-patient-induced pluripotent stem cells, was strongly increased when treated with the well-known GSK-3β inhibitor Kenpaullone [13]. Furthermore, when increased activity and/or upregulation of GSK-3β expression was restored in *Drosophila* [5,14,15] and murine [16,17,18,19] models of ALS, with the treatment of different GSK-3β inhibitors, it was observed a delay in the onset of symptoms, improving motor function and slowing diseases progression.

All of these data, combined with the promising outcomes reported in clinical studies in ALS patients treated with Lithium [20], the first GSK-3β inhibitor identified, suggest that the gain of function of GSK-3β due to the aberrant expression and/or activation could be one of the pathogenic mechanisms of ALS, considering GSK-3β a potential therapeutic target for this devastating neurodegenerative disease.

The main goal of our work is aimed to prove that reduction of the abnormal phosphorylation of TDP-43 with small molecules, inhibitors of specific kinases, could help to recover the homeostasis of this nuclear protein. In that sense, GSK-3β inhibitors emerge as new drug candidates. Tideglusib, a small thiadiazolidindione (TDZD), is an in-house designed non-ATP competitive GSK-3β inhibitor that has shown neuroprotective, anti-inflammatory, and neurogenic properties in different neurodegenerative models [21,22]. Furthermore, good safety, tolerability, and efficacy have been reported in clinical trials for different neurological diseases. In particular, favorable cognitive evolution accompanied by a significant reduction in disease-specific biomarkers in cerebrospinal fluid has been shown in mild AD patients [23]. Moreover, in a phase II trial for Progressive Supranuclear Palsy, Tideglusib-treated patients showed a dose-dependent reduction in brain atrophy progression in comparison with placebo individuals [24]. Tideglusib has also been shown to improve adaptive behavior in children with Autism Spectrum Disorder [25], as well as improvements in neuromuscular symptoms and cognitive function in patients with Myotonic Dystrophy [26], a muscular disease for which this treatment is currently in phase III trials (Clinical trial Identifier: NCT03692312).

On the basis of this background, we have studied the effects of Tideglusib on TDP-43 homeostasis in cellular models of ALS, including immortalized lymphocytes from sporadic ALS patients, as well as in a TDP-43 (A315T) transgenic mouse model. Our results show the efficacy of Tideglusib in the recovery of TDP-43 homeostasis (both phosphorylation and nuclear localization) in ethacrynic acid-treated human neuroblastoma SH-S5Y5 cells and in lymphoblasts from sporadic ALS patients. Moreover, we found that chronic oral treatment with Tideglusib is able to reduce the increased TDP-43 phosphorylation in the spinal cord of TDP-43 (A315T) transgenic mice. Altogether, our data support the repurposing of Tideglusib as a promising therapy for ALS.

## 2. Results

### 2.1. Characterization of Immortalized Lymphocytes from Sporadic ALS Patients

Recently, we demonstrated that lymphoblasts derived from sporadic ALS patients recapitulate the main TDP-43 pathological features such as increased phosphorylation, truncation, and cytoplasmic accumulation [27]. Therefore, we considered this human cell-based model of ALS as a suitable platform for preclinical studies.

**Figure 1:**
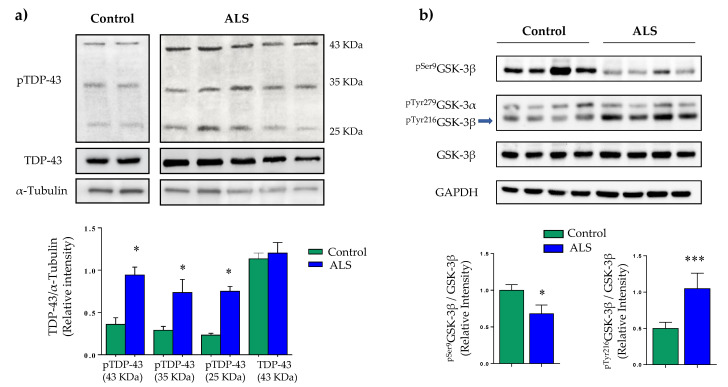
TDP-43 phosphorylation and GSK-3β activity in control and ALS lymphoblasts. Immortalized lymphocytes from control and ALS individuals were seeded at an initial density of 1 × 10^6^ × mL^−1^ in RPMI medium containing 10% FBS. Twenty-four hours after seeding, cells were harvested and processed for Western blotting analysis. (**a**) A representative blot showing levels and phosphorylation status of TDP-43. (**b**) Whole cell extracts were used for the determination of relative levels of phospho-GSK3β (Ser9 and Y216) and total GSK-3β by Western blotting. Densitometric analyses below represent the mean ± SEM of different observations carried out in five cell lines from each group (* *p* < 0.05, *** *p* < 0.001 significantly different from control cells).

Data in Figure 1a confirm and extend previous reports [27,28], by showing increased TDP-43 phosphorylation and protein truncation in ALS lymphoblasts when compared to control cells. Moreover, we have evaluated GSK-3β activity state using specific antibodies against inactive and hyper-active GSK-3β forms, phosphorylated in Ser9 and Tyr216, respectively. Immunoblot analysis showed marked increase in GSK-3β activity as indicated by decreased levels of ^pSer9^GSK-3β together with higher levels of ^pTyr216^GSK-3β (Figure 1b). These results add further support to the idea that GSK-3β may play a pathogenic role in ALS, and, thus, qualify this kinase as potential target for ALS treatment.

### 2.2. Tideglusib Restored TDP-43 Homeostasis in Immortalized Lymphocytes from Sporadic ALS Patients

**Figure 2:**
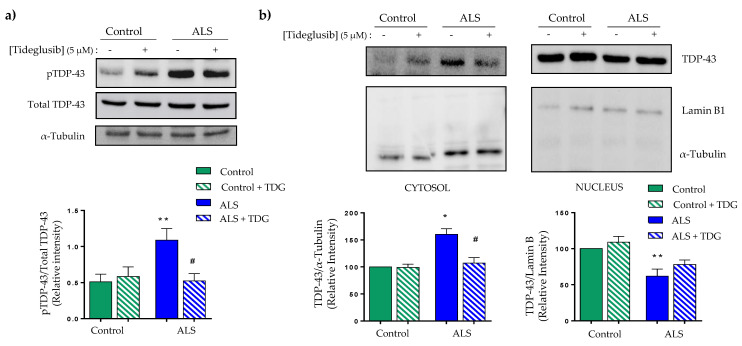
Effects of Tideglusib on TDP-43 phosphorylation and localization in control and ALS lymphoblasts. Immortalized lymphocytes from control and ALS individuals were seeded at an initial density of 1 × 10^6^ × mL^−1^ in absence or presence of Tideglusib (TDG: 5 μM). Twenty-four hours after drug addition, cells were harvested and processed for Western blotting analysis. (**a**) Representative immunoblot showing the effect of Tideglusib decreasing the phosphorylation status of TDP-43 in ALS lymphoblasts. The plot panel below represents the quantifications of the bands of 43 KDa of pTDP-43 normalized by Total-TDP-43. (**b**) After treatment, lymphoblasts were collected and lysed to obtain the cytosolic (left panel) and nuclear (right panel) fractions that were analyzed by Western blotting. α-Tubulin and Lamin B1 antibodies were used as loading and purity control of the cytosolic and nuclear fractions, respectively. A representative experiment is shown. Densitometric analyses represent the mean ± SEM of different observations carried out in five cell lines from each group (* *p* < 0.05, ** *p* < 0.01 significantly different from control cells; ^#^ *p* < 0.05 significantly different from ALS untreated cells).

We next evaluated the effects of Tideglusib treatment on the levels of pTDP-43 in control and ALS lymphoblasts from sporadic ALS patients. As shown in Figure 2a, Tideglusib was able to significantly reduce the aberrant hyperphosphorylation observed in ALS lymphoblasts.

Since it is thought that aberrant phosphorylation of TDP-43 is related with impairment of nucleo-cytoplasmic shuttling of the protein favoring its cytosolic accumulation, we investigated the effects of Tideglusib in subcellular localization of TDP-43 in control or ALS lymphoblasts. Figure 2b shows the results of nuclear and cytoplasmic fractionation experiments. It is shown that TDP-43 accumulates in the cytosolic compartment of ALS lymphoblasts, while levels of nuclear TDP-43 decrease in comparison with control cells (Figure 2b). Tideglusib had no appreciable effects on control cells but was able to efficiently decrease the accumulation of cytosolic TDP-43 in ALS lymphoblasts, observing a trend to restore normal nuclear levels of this protein (Figure 2b).

**Figure 3:**
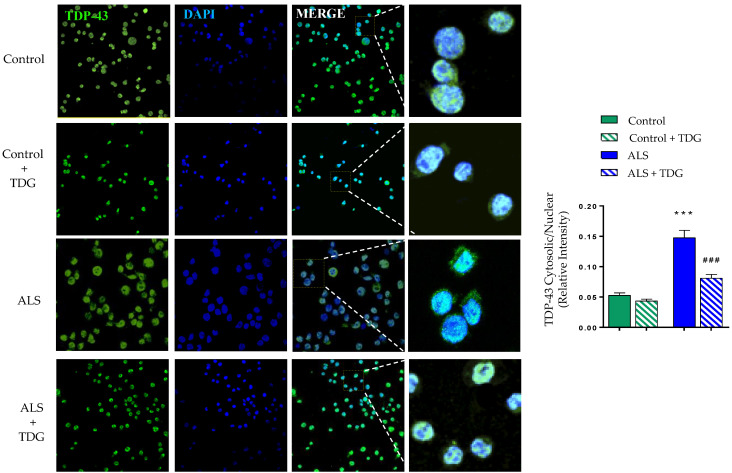
Confocal microscopy analysis of the subcellular localization of TDP-43 after Tideglusib treatment on ALS patients and control lymphoblast. Lymphoblasts (1 × 10^6^ cells × mL^−1^) were incubated with and without Tideglusib (TDG: 5 μM) for 24 h. Confocal laser scanning microscopy was used to examine TDP-43 protein localization. DAPI was used to stain cell nucleus. Relative fluorescence intensity of TDP-43 inside and outside nuclei was determined in two cell lines from each group in at least 45 cells per individual. The plotted values represent the mean ± SEM of quantitative analyses of TDP-43 redistribution (*** *p* < 0.001 significantly different from control cells; ^###^ *p* < 0.001 significantly different from ALS untreated cells).

Similar results were obtained by immunocytochemistry analysis, where treatment with Tideglusib prevented the cytosolic accumulation of TDP-43 on patient’s cells, with no negative effects on control cells (Figure 3).

### 2.3. Effects of Tideglusib on TDP-43 Phosphorylation and Viability in Ethacrynic Acid-Treated Neuroblastoma SH-SY5Y Cells

**Figure 4:**
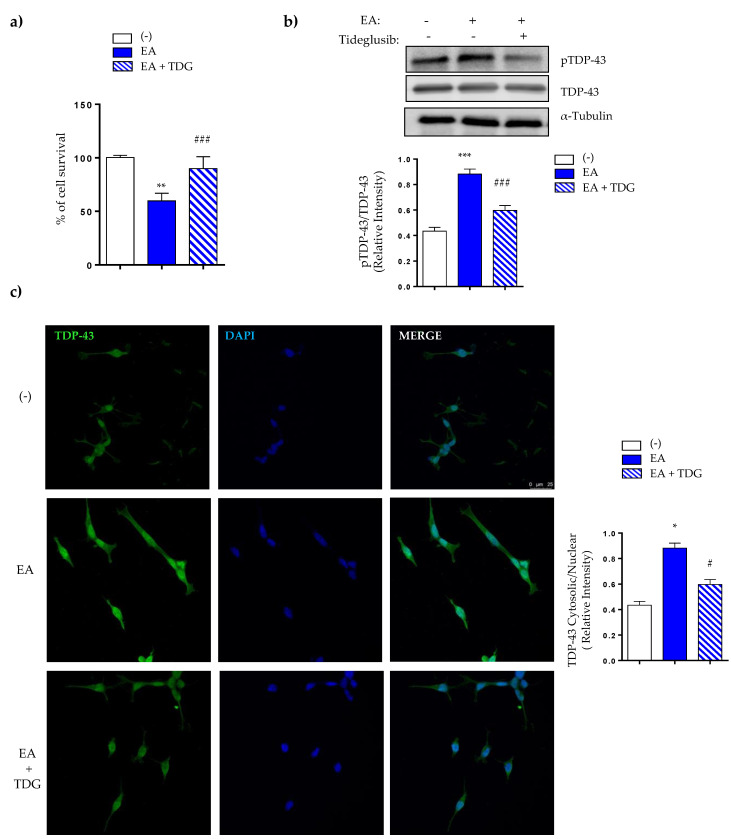
Neuroprotective effects of Tideglusib in ethacrynic acid pre-treated SH-SY5Y neuroblastoma cells. Neuroblastoma SH-SY5Y cells were exposed to 40 μM EA for 12 h in the presence or in the absence of 5μM of Tideglusib (TDG). (**a**) Cell viability after drug treatments was measured by the MTT assay. (**b**) Representative immunoblot showing pTDP-43 protein levels before and after drug treatment. (**a**,**b**), Each data point represents the mean ± SEM of three replications in four different experiments (** *p* < 0.01 significantly different from SH-SY5Y untreated cells; ^###^ *p* < 0.001 significantly different from EA-treated cells). The densitometric data represent the mean ± SEM of three different experiments (** *p* < 0.01, *** *p* < 0.001 significantly different from SH-SY5Y untreated cells; ^###^ *p* < 0.001 significantly different from EA-treated cells). (**c**) TDP-43 protein localization was assessed by confocal laser scanning microscopy. Relative fluorescence intensity of TDP-43 staining inside and outside nuclei were determined on 30 different cells from four separate fields, in each condition. Values are the mean ± SEM. (* *p* < 0.05 significantly different from SH-SY5Y untreated cells, ^#^ *p* < 0.05 significantly different from EA-treated cells).

To validate the results obtained in peripheral cells from ALS patients, we tested the effects of Tideglusib in a neuronal cell model of induced TDP-43 phosphorylation by ethacrynic acid (EA) treatment [29]. For these experiments, SH-SY5Y were preincubated in the absence or in the presence of Tideglusib (5 µM) and then exposed to EA for 24 h. As shown in Figure 4, EA induced a decrease in cell viability (Figure 4a) accompanied by a robust increase in TDP-43 phosphorylation (Figure 4b), in agreement with previous reports [30]. The treatment of SH-SY5Y cells with Tideglusib prevented the cell death caused by EA as detected by the MTT assay (Figure 4a) probably due by decreased of TDP-43 phosphorylation (Figure 4b).

Moreover, Tideglusib was effective in preventing the EA-induced cytosolic TDP-43 accumulation, restoring the homeostasis of the main pathological hallmark of ALS and increasing TDP-43 nuclear localization (Figure 4c).

### 2.4. Tideglusib Does Not Induce Nuclear ß-Catenin Accumulation

**Figure 5:**
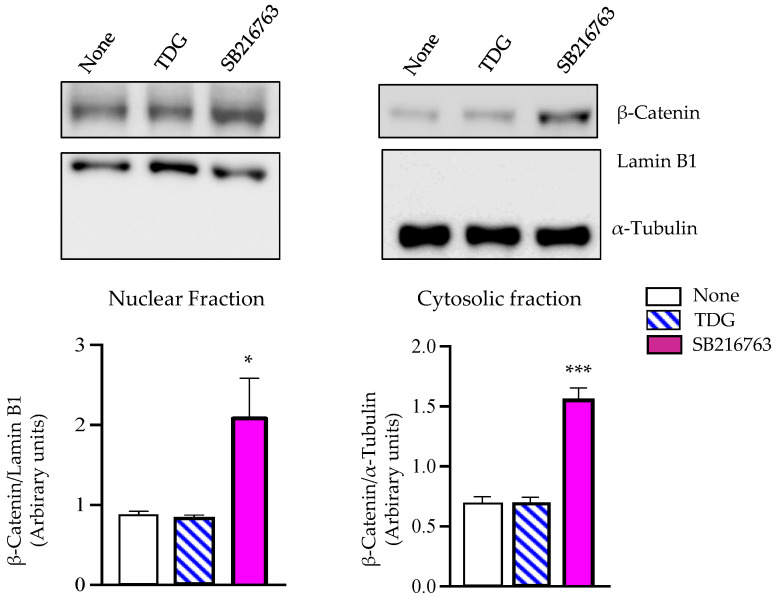
Effects of GSK-3β inhibitors on subcellular localization of β-catenin in human neuroblastoma SH-S5Y5 cells. Cells were incubated for 24 h in the presence or in the absence of 10 µM of Tideglusib (TDG) or SB 216763. After treatment, cells were collected and lysed to obtain the nuclear and cytosolic fractions that were analyzed by Western blotting. Lamin B1 and α-Tubulin antibodies were used as loading and purity control of the nuclear and cytosolic fractions, respectively. A representative experiment is shown. Densitometric analyses represent mean ± SEM of six different experiments (* *p* < 0.05 and *** *p* < 0.001 significantly different from untreated cells).

The involvement of GSK-3β in a number of important signaling pathways including the oncogenic β-catenin pathway [31] raises doubts about the use of GSK-3β inhibitors in clinical settings. Thus, it has been a great challenge to search for suitable GSK-3β inhibitors, able to reduce enzyme activity without interfering this β-catenin signaling. On these grounds, we considered it interesting to explore the effects of Tideglusib on β-catenin subcellular localization, since the non-phosphorylated β-catenin avoids proteasomal degradation, translocates to the nucleus, where it coactivates transcription of different oncogenes [32]. For these experiments, here we have treated human neuroblastoma SH-SY5Y cells, with the non-ATP competitive GSK-3β inhibitor Tideglusib or with the widely used ATP competitive inhibitor SB216783. Localization of β-catenin was assessed by Western blotting in cell fractionated extracts. Figure 5 shows that in cells treated with Tideglusib, β-catenin is not translocated to the nucleus in contrast to the total increased levels and nuclear β-catenin accumulation seen in SB216783-treated cells.

### 2.5. Tideglusib Counteracts TDP-43 Hyperphosphorylation in Prp-hTDP-43 (A315T) Mice

**Figure 6:**
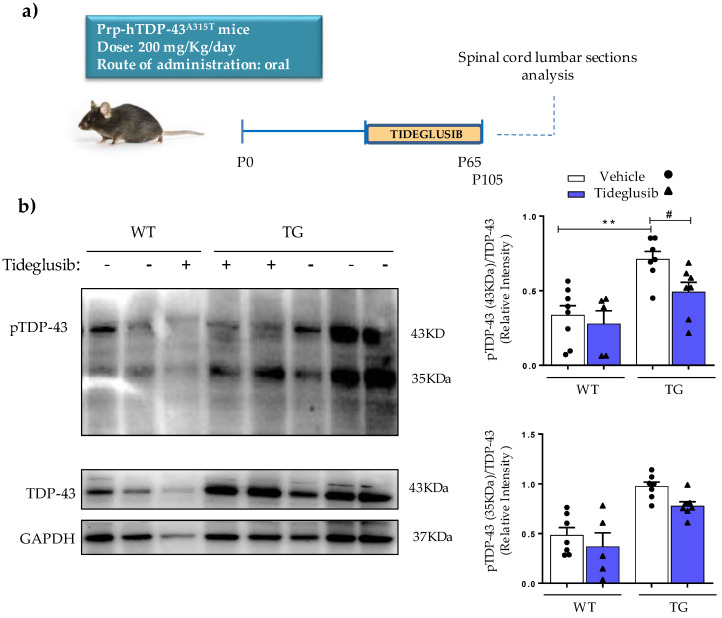
Effects of Tideglusib on levels and phosphorylation status of TDP-43 in the spinal cord of wild-type and transgenic TDP-43 (A315T) mice. (**a**) Experimental design of oral administration of the Tideglusib to wild-type and TDP-43 transgenic mice. (**b**) Effects of Tideglusib treatment on levels and phosphorylation status of TDP-43. Spinal cord lysates from wild-type (WT) and transgenic TDP-43 (TG) mice were used to determine the levels of pTDP-43 and TDP-43. Representative immunoblots are shown. Densitometric analyses represent the ratio of pTDP-43/TDP-43. Values are expressed as mean ± SEM; N ≥ 6 animals in each group (** *p* < 0.01 vs. WT-Veh group; ^#^ *p* < 0.05 vs. TG-Veh group).

Next, we seek to evaluate whether Tideglusib chronic treatment could prevent the enhanced TDP-43 phosphorylation in vivo. To this end, we analyzed the phosphorylation status of TDP-43 in the spinal cord of Prp-hTDP-43 (A315T) transgenic mice, one of the first experimental model of ALS based in TDP-43 mutations [33]. These mice develop motor neuron impairment, neuroinflammation and hyperphosphorylation of TDP-43, as we have recently described [28]. Based on previous clinical trials setup, Tideglusib was orally administrated (200 mg/kg) for 50 days (Figure 6a). As shown in Figure 6b, the chronic administration of Tideglusib resulted in a significant decrease in TDP-43 phosphorylation of both total (43 KDa) and truncated (35 KDa) protein in lysates from lumbar sections of spinal cords of transgenic mice (TG), which reach values close to those found in wild-type mice (WT).

## 3. Discussion

ALS is a fatal motor neuron degeneration disease that lacks an effective treatment, thus, there is a pressing need to search for new therapies. Although ALS pathogenesis has not been fully elucidated yet, it is known that post-translational alterations in TDP-43 protein are present in approximately 97% of ALS patients [1]. Changes in TDP-43 homeostasis associated to ALS include enhanced phosphorylation of TDP-43 at residues Ser 409/410, truncation of the protein and cytosolic TDP-43 accumulation that favors the appearance of protein aggregates [3,4]. Our work aimed at evaluating if reducing the abnormal phosphorylation of TDP-43 could help to recover the homeostasis of this protein, which impacts on multiple signaling pathways. We focus on the modulation of GSK-3β, as it is recognized as one of the main kinases involved in aberrant phosphorylation of TDP-43 [5]. Moreover, alterations in GSK-3β expression and activity have been described in *postmortem* spinal cords and frontal and temporal cortex of ALS patients [7,8,9,10].

We report, here, our results of the treatment of control and sporadic ALS lymphoblasts with Tideglusib, an in-house designed non-ATP competitive GSK-3β inhibitor, that have shown good safety and tolerability in patients with different neurological diseases [23,24,25,26,34]. We have previously demonstrated the usefulness of lymphoblastoid cell lines derived from patients to study TDP-43 homeostasis [27,30] being a suitable platform for preclinical evaluation of potential drug candidates for TDP-43 proteinopathies such as Frontotemporal Dementia (FTD) or ALS [35]. Furthermore, we have shown that by reducing TDP-43 aberrant phosphorylation, we can restore not only TDP-43 homeostasis but also TDP-43 functionality in FTD lymphoblasts [35]. Thus, we have evaluated the influence of Tideglusib in GSK-3β activity and TDP-43 homeostasis in lymphoblasts derived from sporadic ALS patients.

Our data show that GSK-3β inhibition by Tideglusib was accompanied by a significant reduction of the enhanced phosphorylation of TDP-43 protein, observed in ALS lymphoblasts as compared with cells derived from control individuals. In addition, it was found that Tideglusib partially prevented the anomalous cytosolic TDP-43 accumulation in ALS lymphoblasts, thus, recovering TDP-43 homeostasis. These effects of Tideglusib are in accordance with previous work from this laboratory, showing concomitant alterations in TDP-43 phosphorylation and TDP-43 nuclear translocation in either FTLD-TDP and ALS derived lymphoblasts [27,30,35]. Together, these observations suggest that TDP-43 phosphorylation is somehow involved in the control nucleo-cytoplasmic shuttling and reinforce the idea that FTD and ALS linked to TDP-43 pathology represent a disease continuum [36,37].

Despite the fact that ALS-associated changes detected in peripheral cells from patients might not fully reflect those of motor neurons, we have evaluated the potential neuroprotective role of Tideglusib in a neuronal cell model of induced TDP-43 phosphorylation driven by ethacrynic acid, as well as the efficacy of chronic oral administration of Tideglusib in transgenic TDP-43 (A315T) mice, one of the first experimental models of ALS based on mutations in TDP-43 protein [33]. Tideglusib was found to be effective in preventing cell death caused by ethacrynic acid in neuroblastoma cells by inhibiting GSK-3β activity, which resulted in a considerable reduction in TDP-43 phosphorylation. Additionally, this drug was found to significantly reduce the increased TDP-43 phosphorylation in the spinal cord of TDP-43 transgenic mice, demonstrating that at the dose used, Tideglusib was able to reach the central nervous system. The dose of Tideglusib administered to the mice (200 mg/kg daily p.o. for 50 days) is within the range of dosage given to healthy volunteers and AD patients in clinical trials [23,34], in which the good tolerability of this drug was confirmed.

Considering the key role played by GSK-3β in important cellular pathways [38], it was important to rule out potential oncogenic-like effects in future chronic treatments derived from activation of β-catenin signaling. Our results show that Tideglusib appears not to interfere with β-catenin phosphorylation, as we found no β-catenin accumulation in the nucleus of neuronal cells, as opposed to the effect observed after treatment with the ATP-competitive GSK-3β inhibitor SB216763. As reported previously [39], the allosteric, ATP-non-competitive nature of the GSK-3β inhibition induced by Tideglusib allows subtly enzyme modulation avoiding these undesirable effects.

In summary, the results herein presented show neuroprotective effects of Tideglusib both in vitro and in vivo as it restores TDP-43 homeostasis by reducing the aberrant phosphorylation of TDP-43, a consistent feature observed in the cytoplasmic aggregates in affected tissues of about 97% of ALS patients. Given that Tideglusib have shown good safety and tolerability in patients suffering from different neurological diseases, it is proposed that the repositioning of this in-house designed GSK-3β inhibitor could be a valid therapeutic strategy for the treatment of ALS and other TDP-43 proteinopathies. Only results from clinical trials in patients suffering sporadic ALS patients will confirm this therapeutic potential. Recently, a phase II clinical trial protocol has already been approved for this purpose, being expected to begin by the end of the year.

## 4. Materials and Methods

All components for cell culture were obtained from Invitrogen (Barcelona, Spain). Antibodies used in this study were from Santa Cruz Biotechnologies, Heidelgerb, Germany or Proteintech, Manchester, UK and are listed in Table 1.

### 4.1. Cell Lines

Peripheral blood samples were obtained after written informed consent of the patients or healthy individuals listed in Table 2. They were diagnosed as sporadic ALS in the Doce de Octubre Hospital (Madrid, Spain) according to El Escorial criteria [40].

All study protocols were approved by Doce de Octubre Hospital and the Spanish Council of Higher Research Institutional Review Board and are in accordance with National and European Union Guidelines. Establishment of lymphoblastoid cell lines was performed in our laboratory as previously described by infecting peripheral blood lymphocytes with the Epstein-Barr virus [41]. Cells were grown in suspension in T flasks in an upright position, in approximately 8 mL of RPMI-1640 medium (Gibco, BRL) that contained 2 mM L-glutamine, 100 μg/mL penicillin/streptomycin, and, unless otherwise stated, 10% (*v/v*) fetal bovine serum (FBS) and maintained in a humidified 5% CO_2_ incubator at 37 °C. Fluid was routinely changed every two days by removing the medium above the settled cells and replacing it with an equal volume of fresh medium.

Human neuroblastoma (SH-SY5Y) cells were purchased from the European Collection of Cell Cultures (Health Protection Agency, Salisbury, UK), and were propagated in Dulbecco’s Modified Eagle Medium containing L-glutamine (2 mM), 1% non-essential amino acids, 1% penicillin/streptomycin, and 10% fetal bovine serum under humidified 5% CO_2_ and 95% air. On attaining semiconfluency, cells were pre-treated in the absence or in the presence of Tideglusib (5 µM) during 1 h before the addition of ethacrynic acid (40 µM). 24 h later, cultures were processed for cell viability assay. Cell viability was determined by the MTT assay, as previously described [42]. Cell survival was normalized to untreated controls and is presented as a percentage.

### 4.2. Animal Procedures

All the experimental procedures were performed in accordance with the regulations for the use of laboratory animals in the European Commission (2010/63/EU), and with the approval of the ethical committees of the Center of Biological Research (CIB-CSIC) and the regulatory institution (ref. PROEX 066/18). Mice were bred in our animal facilities from initial breeders purchased from Jackson Laboratories (Bar Harbor, ME, USA). Sixty-five-days-old male Prp-hTDP-43(A315T) transgenic mice and non-transgenic littermate siblings were used. As described previously [33], the presence or absence of *TARDBP* transgene amplification, containing A315T mutation, confirmed mice genotypes. Wild-type and transgenic mice were identified with ear punching codes and randomly allocated to the different treatment groups. Tideglusib was orally administered at 200 mg·kg^−1^·day^−1^ for 40 days (P105). All the mice were housed at 22 ± 1 °C with natural light/dark cycles (12 h). High fat jelly food (DietGel Boost, ClearH20, Portland, ME, USA), specific for TDP-43 transgenic mice [43], and water were available *ad libitum*.

### 4.3. Immunoblotting Analysis

Cells were collected by centrifugation, washed with PBS and total protein extracts were obtained by lysing them as previously described [30]. Nuclear and cytosolic fractions were obtained using the Subcellular Protein Fractionation Kit, (Cat#78840, Thermo Fisher Scientific, Madrid, Spain) following the manufacturer’s instructions. Lamin B1 and α-Tubulin were used as markers for nuclear and cytosolic fractions, respectively. Mouse spinal cords were lysed in RIPA buffer supplemented with a protease and phosphatase inhibitor cocktail (Roche, Mannhein, Germany). The protein content of the extracts was determined by the Pierce BCA Protein Assay kit (ThermoFisher, Madrid, Spain). Equal amounts of proteins were resolved by SDS–polyacrylamide gel electrophoresis. Proteins were then transferred to polyvinylidene fluoride (PVDF) membranes and immunodetected, as previously described [30]. The primary antibodies used are listed in Table 1. Signals from the primary antibodies were amplified using species-specific antisera antibodies conjugated with horseradish peroxidase and detected with a chemiluminescent substrate detection system ECL (Bio-Rad, Alcobendas, Madrid, Spain). Relative band intensities were quantified using a ChemiDoc station with Quantity One 1D analysis software (Bio-Rad Laboratories, Madrid, Spain) and normalized by those of GAPDH, α-tubulin, or Lamin B1.

### 4.4. Immunofluorescence

Lymphoblasts (1 × 10^6^ × mL^−1^) or SH-S5Y5 cells (300,000) were fixed for 30 min in 4% paraformaldehyde in PBS and blocked and permeabilized with 0.5% Triton X-100 in PBS–0.5% BSA for 60 min at room temperature. Lymphoblasts were attached to poly-L-lysine-coated coverslips using the Cytospin centrifuge at 700 rpm for 7 min. After being fixed, cells were incubated overnight with anti-TDP43 polyclonal antibody. After removing the primary antibody, cells were washed with PBS and incubated with Alexa Fluor 488-conjugated anti-rabbit antibody. For nuclear staining, the preparations were mounted on ProLong^®^ Gold Antifade Reagent with DAPI (Thermo Fisher) allowing nuclear visualization. High-resolution images were acquired for ∼ 45 cells per group in n = 2 independent experiments using the LEICA TCS-SP5-AOBS confocal microscope system (Heidelberg, Germany). Quantification of TDP-43 was performed using Image J software. Data is expressed as the ratio of the fluorescence intensity of cytosolic TDP-43 vs. the intensity of the fluorescence of the nuclear protein.

### 4.5. Statistical Analysis

Statistical analyses were performed with Graph Pad Prism 9. All the statistical data are presented as mean ±standard error of the mean (SEM). Normality was checked with the Shapiro–Wilk test. Parametric tests were, therefore, used in the statistical analysis. Statistical significance was estimated by Student’s *t*-test or by analysis of variance (ANOVA) followed by the Fisher’s LSD test for multiple comparisons. A value of *p* < 0.05 was considered significant.

## Figures and Tables

**Table 1:** List of antibodies used.

Antibody	Source	Catalog Number	Dilution
Lamin B1	Santa Cruz Biotechnologies	sc-6217	1:1000
α-Tubulin	Sigma	L7543	1:1000
pTDP-43 (Ser409/410)	Proteintech	22309-1-AP	1:500
TDP-43	Proteintech	10782-2-AP	1:1000
GAPDH	Santa Cruz Biotechnologies	sc-25778	1:500
p-GSK-3β(Ser9)	Santa Cruz Biotechnologies	sc-11757)	1:500
p-GSK3β(Y216)	Santa Cruz Biotechnologies	sc-81496	1:500
GSK-3β	Santa Cruz Biotechnologies	sc-9166	1:500
β-Catenin	Santa Cruz Biotechnologies	sc-7199	1:500

**Table 2:** Demographic and clinical characteristics of subjects included in this study.

	Control (N = 5)	ALS (N = 5)
Gender (M/F)	(2/3)	(2/3)
Family history	No	No
Age (±SD)	62 ± 7	65 ± 1
Site of onset		
Bulbar	NA	4
Limb	NA	1

Control: Individuals without sign of neurological degeneration, NA: not applicable. All patients were negative for *SOD1, TARDBP,* and *C9orf72* mutations.

## Data Availability

The datasets analyzed during the present study are available from the corresponding authors on reasonable request.
